# Micro-Dumbbells—A Versatile Tool for Optical Tweezers

**DOI:** 10.3390/mi9060277

**Published:** 2018-06-01

**Authors:** Weronika Lamperska, Sławomir Drobczyński, Michał Nawrot, Piotr Wasylczyk, Jan Masajada

**Affiliations:** 1 Department of Optics and Photonics, Faculty of Fundamental Problems of Technology, Wrocław University of Science and Technology, Wybrzeże Wyspiańskiego 27, PL50370 Wrocław, Poland; slawomir.drobczynski@pwr.edu.pl (S.D.); jan.masajada@pwr.edu.pl (J.M.); 2 Photonic Nanostructure Facility (PNaF), Faculty of Physics, University of Warsaw, ul. Pasteura 5, PL02093 Warszawa, Poland; michal.nawrot@fuw.edu.pl (M.N.); pwasylcz@fuw.edu.pl (P.W.); 3 Wellcome/EPSRC Centre for Interventional and Surgical Sciences, University College London, Gower St, London WC1E 6BT, UK

**Keywords:** optical tweezers, optical trapping, viscosity, direct laser writing, 3D lithography, two-photon polymerization, micro-tool

## Abstract

Manipulation of micro- and nano-sized objects with optical tweezers is a well-established, albeit still evolving technique. While many objects can be trapped directly with focused laser beam(s), for some applications indirect manipulation with tweezers-operated tools is preferred. We introduce a simple, versatile micro-tool operated with holographic optical tweezers. The 40 µm long dumbbell-shaped tool, fabricated with two-photon laser 3D photolithography has two beads for efficient optical trapping and a probing spike on one end. We demonstrate fluids viscosity measurements and vibration detection as examples of possible applications.

## 1. Introduction

**Figure 1:**
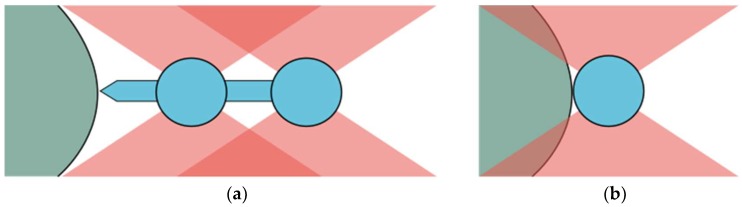
(**a**) For probing with a micro-dumbbell, the examined object (green) is not exposed to the focused trapping beams, whereas for a single bead (**b**) a fraction of the beam illuminates the object and may cause unwanted effects.

Optical tweezers use the phenomenon of optical trapping to manipulate small objects in microscale. Tightly focused laser beam becomes an optical trap—in the vicinity of the focus an interplay of attractive and repulsive forces results in a trapping potential. Several requirements need to be fulfilled for an object to be trapped. These include partial transparency, weak absorption at the trapping wavelength and the refractive index exceeding the one of the surrounding medium. To circumvent these limitations, instead of trapping the object itself, micro-tools can be held and operated by optical tweezers. Since spherical shapes provide the most stable trapping, the so-called micro-beads of various sizes and materials are employed to this end. They can be used as a probe [1], a handle [2] or a marker [3] and their simple shape is both an advantage and a limitation. Various micro-tools have been demonstrated [4,5,6,7,8,9] that expand the concept of optical trapping and manipulation beyond the simplest micro-beads. Here we propose a new one: a micro-dumbbell, made of two connected beads and a spike (Figure 1a). Due to their simplicity micro-dumbbells can be manufactured in large volumes with standard lithographic technique and their parts can be rescaled according to the specific needs. The energy density of the focused trapping beam can be very high and may have uncontrolled influence on the sample (Figure 1b). The front spike enables exerting force on the objects under examination (e.g., cells) without exposing them directly to the laser light. The micro-dumbbells are controlled using the two beads trapped in laser beams. Since the entire trapping beams go through these two beads, the measurement methods and data analysis is the same as in the case of a single beam.

## 2. Materials and Methods

### 2.1. Optical Micro-Dumbbell

**Figure 2:**
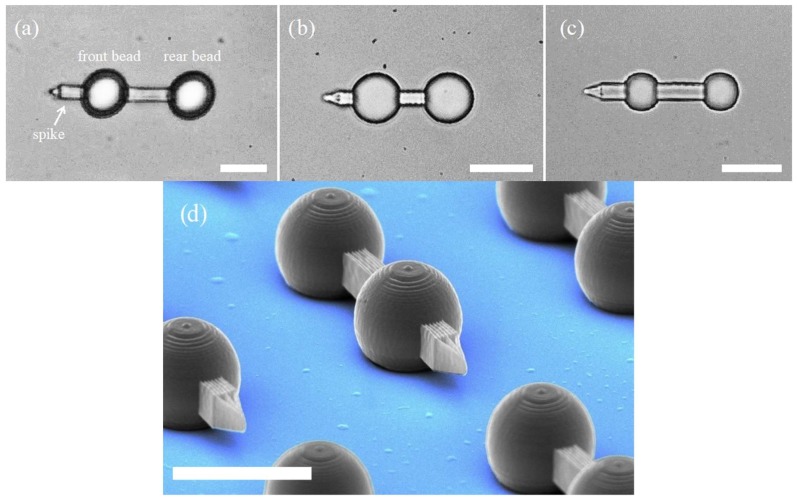
Optical microscope images of three micro-tool designs with different bead diameters Ø and total lengths L; (**a**) Ø = 10 µm, L = 37 µm; (**b**) Ø = 8 µm, L = 25 µm; (**c**) Ø = 6 µm, L = 26 µm. (**d**) Scanning electron microscope (SEM) image of the micro-tools on the glass substrate (with computer-added colors) shows the limits in resolution of the 3D laser photolithography. Scale bars: 10 µm.

A micro-dumbbell consists of two spherical beads with a bar between them and a spike on one end (Figure 2). In Figure 2a–c three different designs of the micro-tool are presented. They vary in the size of the bead, the overall proportions and the total length. All three types proved to work well, which indicates that the size of the tool can be adjusted to specific needs, with the limits imposed by the fabrication technology. The beads are trapped with optical trapping beams, one for each bead. Both traps have the same stiffness and the two beams are focused at the same depth in the specimen. By moving both traps simultaneously, the tool can be moved around and rotated in 3D space. Once trapped, the tool aligns itself in the horizontal position (i.e., parallel to the coverslips) and floats horizontally even in the absence of optical traps. That means that the trapping process is easy and keeping the tool in horizontal position requires no additional forces.

The spike has two main features: due to the small area of its tip, the pressure exerted on objects under study (e.g., erythrocyte membrane [10,11]) can be much higher than in the case of a single bead. As the probing tip is at a distance from the trapping targets (the beads), the illumination of the object under study can be reduced, which is important for highly absorbing and/or sensitive (e.g., biological) specimens.

### 2.2. Fabrication

**Figure 3:**
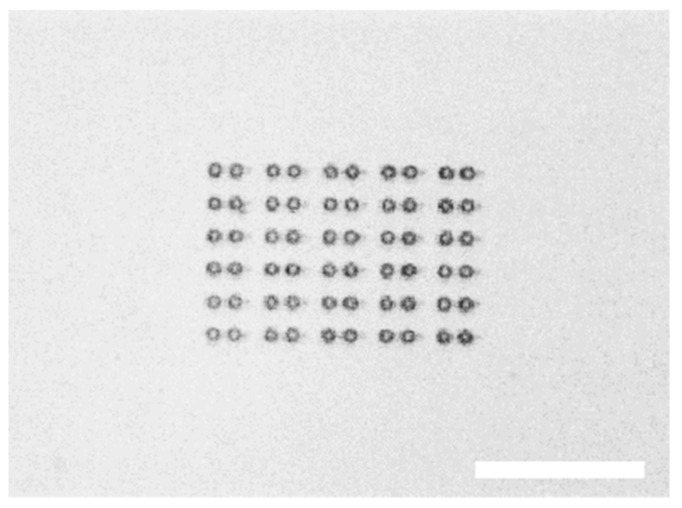
Optical microscope photograph of an array of 30 micro-tools printed on a glass substrate (microscope coverslip). Printing the entire matrix of micro-tools makes it easier to locate the tools on the glass slide after they are detached and float in a water-filled chamber. Scale bar: 100 µm.

The tool shape was designed in a CAD software and the micro-dumbbells were manufactured with 3-dimensional two-photon laser lithography [12] (*Photonics Professional*, Nanoscribe) [13]. Several identical micro-tools were laser-printed in a UV-curable photoresist (IPL, Nanoscribe) on a glass coverslip, the unsolidified resin was removed in the isopropyl alcohol and the plate was dried with compressed nitrogen and air. A single sample contained a matrix of 20 to 30 dumbbells (Figure 3). Once the fabrication process was finished, the micro-tools remained attached to the substrate and could be stored for a long time (a few months) before being used.

The substrate glass coverslip (0.17 mm thickness) was first coated with a several hundred nanometer thick layer of polyvinyl alcohol (PVA) that acts as a separation layer—once the micro-tools are printed and a drop of water is deposited on the glass, the PVA layer dissolves, releasing the tools. Very thin PVA layer guarantees low (below 0.1%) PVA concentration in water. Without the PVA separation layer the tools can be detached mechanically from the glass surface with a needle tip, but such operation provides little control and damages many of them.

Finally, a 1 mm high chamber was added on the glass plate and the micro-tools were sandwiched between two coverslips, immersed in 20 µL of water. As the separation between the coverslips (the chamber height) was much larger than micro-tool size, the effects associated with the proximity of the glass surfaces could be neglected.

### 2.3. Experimental Setup

**Figure 4:**
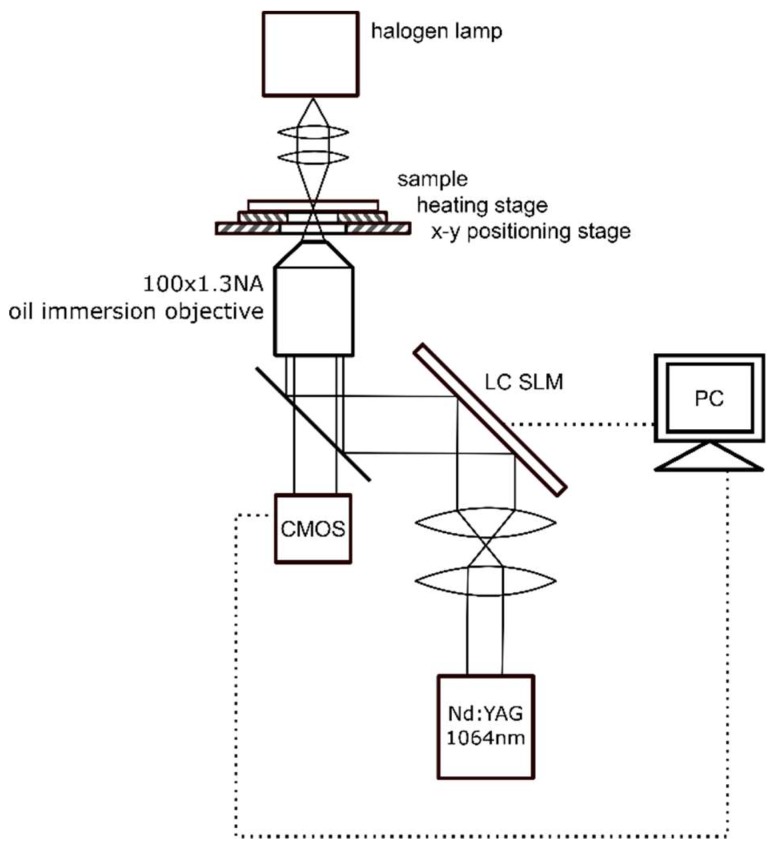
Schematic of the holographic optical tweezers setup.

**Figure 5:**
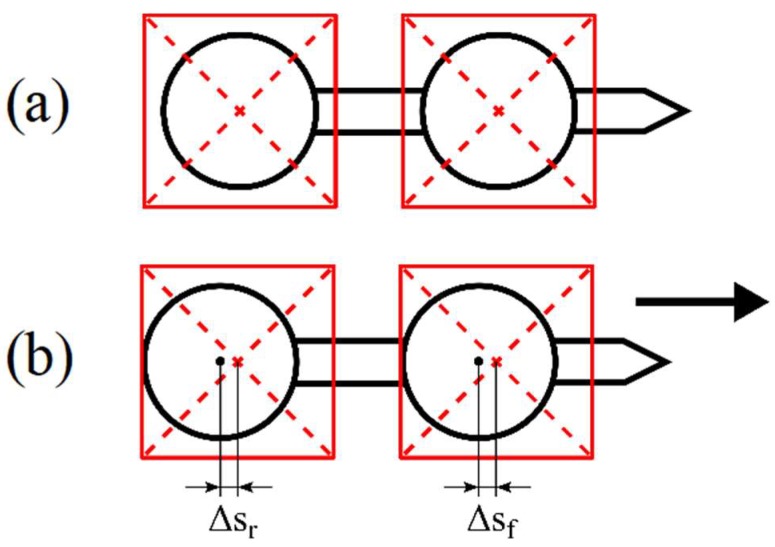
Trapped bead displacement with respect to the trapping laser beams. For a stationary tool (**a**), the trap centers (red squares) overlap with the centers of the beads. When the traps move and pull the tool (**b**), the displacements between the trapping beams and the beads centers Δs_f_ and Δs_r_ may be used to measure the drag forces. Further in the paper ‘Δs’ is used to denote any displacement since it is clear from the context whether it refers to the front or rear bead.

The holographic optical tweezers setup [14] is schematically presented in Figure 4. It is based on the inverted microscope (Olympus IX71) and the Nd:YAG laser (1064 nm, 4 W, CW, Laser Quantum) as a trapping source. The laser beam is collimated and illuminates an SLM matrix (Spatial Light Modulator, HoloEye-Pluto). After reflection from the SLM, the beam is tightly focused with a high-numerical aperture oil-immersion objective (100×, NA = 1.3, Olympus UPlanFL). The SLM acts as a dynamic, programmable diffraction grating and generates two optical traps in a sample plane—one for each bead of the micro-tool. A heating stage maintains the sample temperature, in particular during the viscosity measurements. The microscope image is recorded with a CMOS camera (Mikrotron Inspecta) working in a high-speed mode and the location of the traps as well as the position of the trapped beads is registered and analyzed. The displacement between the trapped bead and the trap centers (Δs in Figure 5) is a measure of the external force acting upon the bead, and can be used to determine, for example, the resistance of the medium.

The micro-tool can be moved in liquid in two ways: either the trapping beams are moved by changing the hologram pattern displayed on the SLM, or the laser beams are kept stationary and the chamber moves. The major disadvantage of the first approach stems from the limited response time of the liquid-crystal molecules in the SLM [15]. During the time needed for the molecules to rearrange, the SLM state and thus the trapping beam shape and position are, to a certain extent, random [16,17].

The other method uses the microscope x-y translation stage, driven either manually or with a piezo-drive. Here the trapped tool motion is smoother, but the final velocity estimation is less accurate. In the following experiments the latter technique was used.

## 3. Results

### 3.1. Streamlined Shape

**Figure 6:**
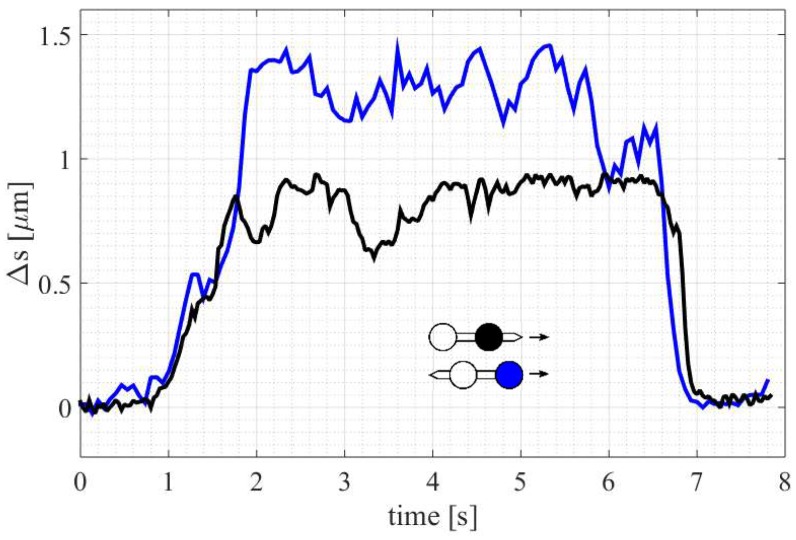
Drag force measurement for the micro-tool moving in two directions. Measured displacement of the front bead for the forward (black) and backward (blue) orientation of the tool. The tool velocity was increasing for the first 2 s, then kept constant and finally reduced to zero during the last 1.5 s.

The first experiment looked into the dependence of the drag on the micro-tool motion direction. The displacements from the trap centers (Δs) of both beads were recorded for the ‘forward’ (probing spike in the front) and ‘backward’ (spike at the back) directions (Figure 6). The results show that the drag force exerted on the front bead increased more than twice in the second case. The streamlined shape of the dumbbell is effective in reducing the resistance of motion. One can conclude that adding a small spike in front of the bead (see Figure 2) remarkably reduces the drag forces.

### 3.2. Fluid Viscosity Comparison

**Figure 7:**
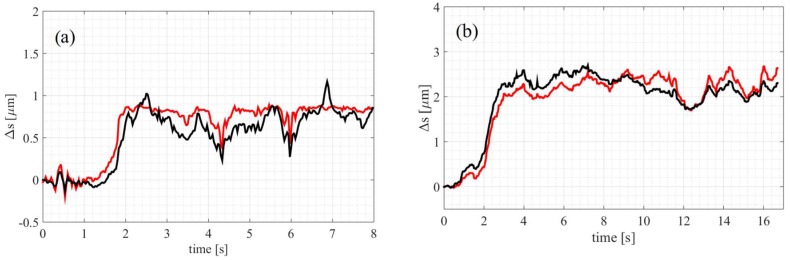
Fluid viscosities can be compared by measuring the trapping beam-bead displacement in an accelerating tool. The displacements of the front (black) and rear (red) beads for the tool moving in water (**a**) and the 30% glycerol solution (**b**). At the beginning, the velocity increases (first 2 and 3 s, respectively) and then remains constant.

The trapping beam-bead displacement can be used for fluid viscosity characterization [1,18]. The procedure involves dragging the tool through the medium of an unknown viscosity and then through a reference medium. Water was used as a reference medium with the viscosity of 0.853 mPa·s (27 °C). The medium under examination was 30% glycerol solution (*v*/*v*) with the previously measured viscosity equal 2.43 mPa·s (27 °C) [19]. Both beads were trapped and the micro-tool was set into motion with the maximum velocity for which the beads do not yet escape from the traps. This happens when the external force on the trapped object (in our case, the drag force) exceeds the trapping force. The trajectories of the beads for the reference and measured media are shown in Figure 7. The mean displacement Δs in the ‘plateau’ regions is ~0.8 µm for water and ~2.5 µm for glycerol solution. This difference can be used to compare the two fluids’ viscosities with only several microliters samples.

### 3.3. Vibrations Detection and Measurements

**Figure 8:**
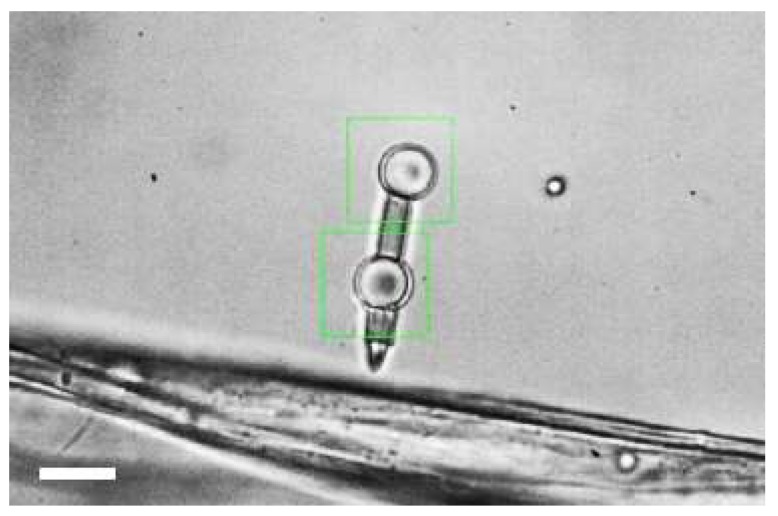
A micro-tool with the probing spike touching a cotton fiber. The thickness of the fiber is about 20 µm. Scale bar: 10 µm.

In this experiment the micro-tool was used to detect vibrations of a cotton fibers suspended in water. The tool spike was put in contact with a 20 um diameter fiber (Figure 8). There were three main factors affecting the motion of the objects in the sample. First, thermal noise of approximately uniform distribution (white noise). Second, mechanical vibrations, especially originating from the cooling system, i.e., the fan (≈77 Hz). Third, the refresh rate of the SLM (60 Hz), accompanied by the so-called addressing rate—the liquid crystal molecules in the SLM are addressed at a number of refresh rates (120 Hz and overtones) [20,21]. The refresh and address rate are experienced only by the objects directly illuminated by the laser beam– in this experiment, the trapped micro-tool beads, but not the fiber.

**Figure 9:**
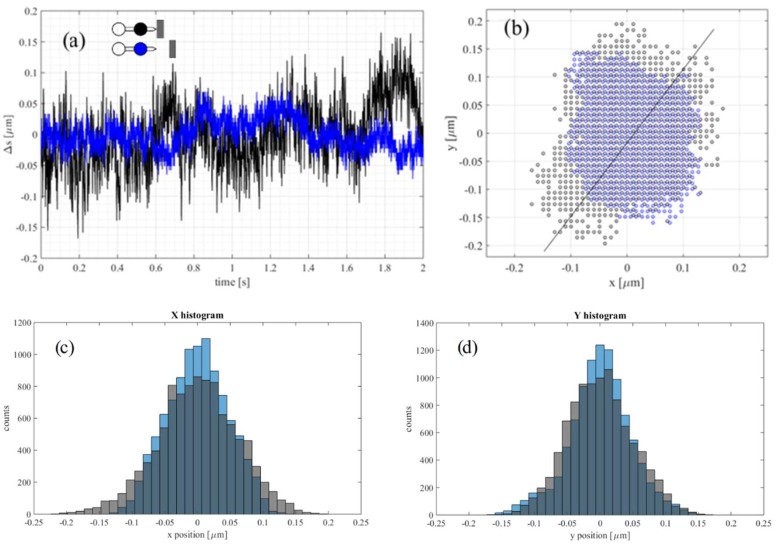
Vibrations measurements in microscale. (**a**) Displacement of the front bead for the trapped micro-tool in contact (black) and not in contact (blue) with the cotton fiber. The two measurements were performed in different two-second intervals and are presented on the same plot for comparison; (**b**) corresponding position of the front bead in the x-y plane (the sample plane) measured every 0.2 ms. The alignment of the micro-dumbbell in the x-y plane corresponds to the one in Figure 8. A solid black line indicates the direction along which histograms in (**c**,**d**) were calculated; (**c**,**d**) histograms of the x and y positions of the bead along the line from (**b**). Blue bins correspond to blue points, gray bins to black points and the dark grey color is the overlapping region.

The position of the front bead was recorded with sampling rate of 5000 fps with the probing tip in contact with the fiber and then without. The trajectories for the two cases (‘contact’ and ‘no contact’) are presented in Figure 9a. There is a visible difference in the amplitude of fluctuations for the two cases—when the probing tip was in contact with the fiber, the displacements of the bead were over twice as large as for the tool with no contact with the fiber.

**Figure 10:**
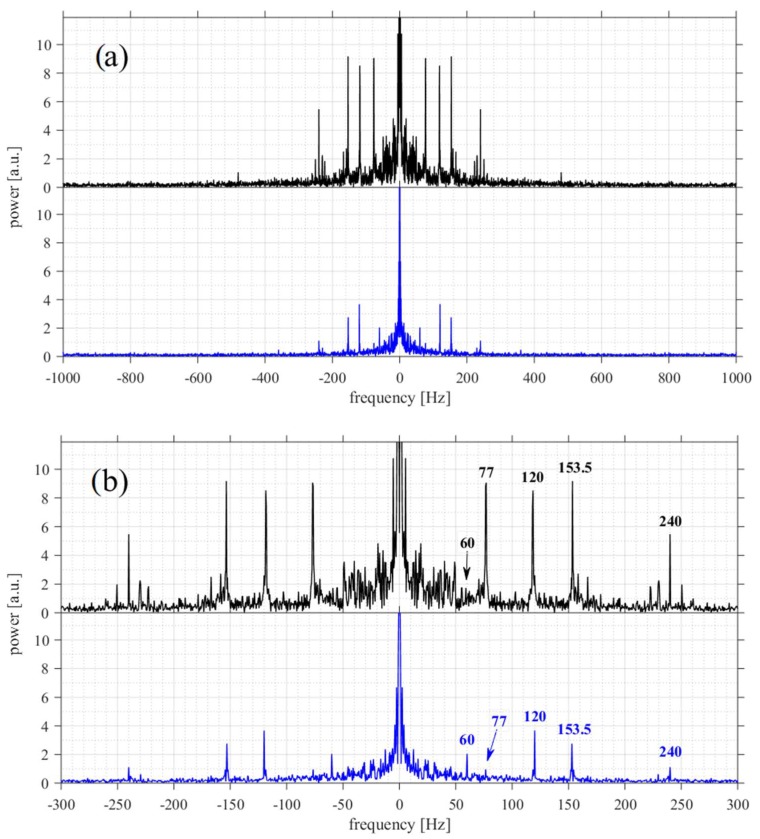
Fourier spectra of the two signals shown in Figure 8a, corresponding to the ‘contact’ (black) and ‘no contact’ (blue) cases. Main frequency peaks in the black plot are at 77, 120, 153.5, 240, 480 Hz. Main frequency peaks in the blue plot are at 60, 120, 153.5, 240 Hz. Sampling frequency was 5000 Hz, we show only the range of ±1000 Hz in (**a**) and ±300 Hz in (**b**) for clarity.

Fourier transform of the two recordings (Figure 10) reveals another interesting feature. Although in both measurements some common frequency peaks were detected, for the tool in contact with the fiber their amplitude is larger and more overtones are visible. The 120 Hz and 240 Hz peaks are two and almost six times larger, respectively. This effect is due to the nonzero force at which the tool is pushed against the fiber. As a result, the equilibrium state for the bead is off the trap center and the oscillation amplitude increases. This is also visible in Figure 9b, where the positions of the front bead in the x-y plane (sample plane) are plotted for the two configurations. The circular pattern for the freely floating tool indicates the Brownian character of the bead motion. For the tool in contact with the fiber, the pattern is stretched along the line parallel to the tool axis.

Since the cotton fiber touches the microscope slide, the mechanical vibrations are transferred to it. In contrast, for the freely floating micro-tool these vibrations are damped by water. This results in the strong 77 Hz peak (the cooling fan frequency) being almost ten times weaker in the ‘no contact’ mode. Its first overtone, at around 154 Hz, is three times higher. Other low frequency peaks in the contact case result from unidentified sources of mechanical vibrations in the laboratory and its neighborhood.

## 4. Discussion

We have designed, fabricated and characterized a micro-dumbbells tool operated with holographic optical tweezers, bringing new possibilities to the optical manipulation in microscale. Due to the simple design of our micro-tool, its oscillations as well as the force exerted on the spike can be measured using the standard procedures for the polystyrene bead tracking [22,23,24]. Two applications of the micro-tools were demonstrated: fluid viscosity characterization in a small volume sample, and detecting vibrations of micro-objects.

Tracing the tool position with sufficiently high sampling frequency and performing Fourier transform of the data allowed us to detect several vibration modes of a cotton fiber suspended in water. This technique can be directly adopted for self-vibrating specimen such as bacteria [25] or yeast [26], as with the micro-dumbbells the measured object is not illuminated with the trapping beams. The sharp sensing spike can be used to exert high pressure, thus opening up a way to determine elastic properties of micro-objects. However, for such applications, optical tweezers with galvanic mirrors for beam steering, offering higher trap stiffness, are needed. In more elaborate experiments, the spike can be covered with a metal layer. While illuminated with a laser beam, it will become a heating element useful for various studies on thermal effects. It can be also functionalized in any other possible way to meet the specific measurement needs.
